# Time-frequency component analysis of somatosensory evoked potentials in rats

**DOI:** 10.1186/1475-925X-8-4

**Published:** 2009-02-09

**Authors:** Zhi-Guo Zhang, Jun-Lin Yang, Shing-Chow Chan, Keith Dip-Kei Luk, Yong Hu

**Affiliations:** 1 Department of Orthopaedics and Traumatology, The University of Hong Kong, Pokfulam, Hong Kong, PR China; 2 Department of Orthopaedics, The 1st Affiliated Hospital of Sun Yat-Sen University, Guangzhou, PR China; 3 Department of Electrical and Electronic Engineering, The University of Hong Kong, Pokfulam, Hong Kong, PR China

## Abstract

**Background:**

Somatosensory evoked potential (SEP) signal usually contains a set of detailed temporal components measured and identified in a time domain, giving meaningful information on physiological mechanisms of the nervous system. The purpose of this study is to measure and identify detailed time-frequency components in normal SEP using time-frequency analysis (TFA) methods and to obtain their distribution pattern in the time-frequency domain.

**Methods:**

This paper proposes to apply a high-resolution time-frequency analysis algorithm, the matching pursuit (MP), to extract detailed time-frequency components of SEP signals. The MP algorithm decomposes a SEP signal into a number of elementary time-frequency components and provides a time-frequency parameter description of the components. A clustering by estimation of the probability density function in parameter space is followed to identify stable SEP time-frequency components.

**Results:**

Experimental results on cortical SEP signals of 28 mature rats show that a series of stable SEP time-frequency components can be identified using the MP decomposition algorithm. Based on the statistical properties of the component parameters, an approximated distribution of these components in time-frequency domain is suggested to describe the complex SEP response.

**Conclusion:**

This study shows that there is a set of stable and minute time-frequency components in SEP signals, which are revealed by the MP decomposition and clustering. These stable SEP components have specific localizations in the time-frequency domain.

## Background

Somatosensory evoked potential (SEP) is the electrical response of the central nervous system to an electrical stimulation of a peripheral nerve. It has been widely used in electrophysiological diagnosis and intraoperative neurophysiology monitoring [1-4]. Previous studies demonstrated that there are a series of detailed temporal components in SEP as well. They reflect sequential activation of neural structures along the somatosensory pathways [3-6]. These detailed temporal components of short durations and small amplitudes are generally identified by measuring latencies of a set of small onsets, peaks and notches in time domain.

Recently, measured SEP signals in frequency domain and time-frequency (t-f) domain were noticed by researchers and were suggested as important indicators of spinal cord injury [7-12]. Time-frequency analysis (TFA) of SEP recording is capable of revealing stable and easily-identifiable SEP characteristics in t-f domain and presented rapid changes when deficits happened in spinal cord function [7,8]. More precisely, a SEP signal can show a distinct peak in its time-frequency distribution (TFD). Feature extraction is based on the measurement of parameters associated with the peak, such as peak power, peak time and peak frequency [9-12]. This observation motivated us to find out detailed SEP time-frequency components using TFA methods. Unlike the temporal components measured in time domain, a t-f component is measured in t-f domain and can be clearly described by a set of time and frequency parameters.

Although the main SEP t-f component can be identified from the prominent peak in TFD, other detailed t-f components (hereinafter called "subcomponents") can hardly be revealed from the TFD. Possible reasons include the huge dominance of the main t-f component, the minuteness of t-f subcomponents and the low t-f resolution of TFA methods in some previous studies [8-12]. By adjusting the window function, the time or frequency resolution of TFA can be improved, but they cannot be simultaneously improved due to the time-frequency uncertainty principle, which implies a higher time resolution at the expense of a lower frequency resolution and vice versa. In [13], a multi-resolution wavelet analysis of SEP was proposed and it decomposed the signals into a series of coarse and detailed t-f components with the help of scaling and wavelet functions. This method provided a new way (time-frequency decomposition) to analyze SEP signals, but the wavelet analysis could not offer a time-frequency parameter description for the decomposed components, so it is difficult to characterize the t-f components and establish an objective standard to evaluate the SEP.

To overcome the limitations of wavelet analysis and other TFA methods, a high-resolution TFA algorithm, the matching pursuit (MP), will be adopted in this paper to analyze SEP signals. The MP algorithm was first introduced by Mallat and Zhang [14], and its basic idea is to decompose a signal into a series of t-f components from a very large and redundant dictionary. By adaptive approximation, the MP algorithm can offer a higher t-f resolution than wavelet analysis and other TFA methods. Besides its high resolution, the MP algorithm is able to provide a straightforward parameter description of decomposed components including their locations in t-f domain. Furthermore, the MP method is very robust in the presence of heavy background noise. Additive Gaussian white noise with a signal-to-noise ratio (SNR) -3dB, which means that the addition of noise has twice the power of the signal, does not critically influence the t-f positions of the t-f components [15].

Having all these advantages, the MP algorithm has attracted increasing interests from biomedical researchers and its applications to various biomedical signals including electroencephalography (EEG) [15,16], otoacoustic emission (OAE) [17,18], visual evoked potential (VEP) [19,20] and heart rate variability (HRV) [21], have been reported. In our study, the MP algorithm will be employed to decompose SEP signals in rats into a number of t-f components with certain parameter (feature) description.

Among the decomposed t-f components, we aim to identify stable SEP components useful for understanding the underlying physiological mechanisms of SEP and evaluation of somatosensory conduction. However, spurious and unstable t-f components may also be generated by the MP algorithm and they will influence accurate identification of stable components to some extent. The spurious components are mainly caused by electrophysiological noise, while unstable components are mainly due to some physiological causes, as concluded in [4]. To find out stable SEP components with similar parameters (features), an unsupervised clustering technique based on the probability density function (PDF) estimation of the t-f component parameters was performed [22,23]. A stable SEP component should have high joint PDF values in the high-dimensional space defined by the component parameters. The joint PDF of these parameters will be calculated by kernel density estimation and clusters are identified as local maxima in the PDF. If the decomposed t-f components in one cluster occur in a majority of SEP signals under study, they will be classified as the same kind of stable t-f component.

The purpose of this study is to establish a practical method to extract detailed SEP components and thereby identify stable SEP components. Experimental results on rat SEP recordings show that a set of stable SEP components can be identified using the proposed MP decomposition and joint PDF estimation.

## Methods

### Experimental Procedure and Data Collection

The same SEP data set tested in [9] is used in this study. Twenty-eight mature rats weighing between 260 and 280 grams were used. All the experimental procedures were performed under intravenous pentobarbital (0.05 mg/g) anaesthesia augmented by local 1% xylocaine infiltration. Additional pentobarbital was given at intervals and in amounts established in non-curarized rats to assure adequate anaesthesia.

To elicit cortical SEP, a constant current stimulator was used to apply a 5.1 Hz square wave 0.2 ms in duration to the hind paw. The current was set to cause mild twitch of limbs (3–10 mA). The cortical SEP was recorded from the skull at Cz-Fz. The signal was amplified 100,000 times with two amplifiers (SCXI-1120, National Instruments Co, TX, USA). Bandpass filtering between 2 Hz and 2000 Hz was used. All the SEP signals were acquired with a data acquisition card (DAQcard-1200, National Instruments Co, TX, USA) at 12 bit resolution and a sampling rate of 5000 Hz. To obtain a good SNR for the SEP signals, a total of 100 SEP responses were averaged for each trial.

In this study, all the following signal processing programs were developed in MATLAB environment (version 7.0, Mathworks, MA, USA) using the Pentium 4 PC platform (3.2G Hz, 1G bytes RAM).

### Matching Pursuit Decomposition

Given a discrete-time signal *x*(*n*), the MP method decomposes the signal *x*(*n*) into a linear combination of basic functions {*g*_0_(*n*), *g*_1_(*n*), ..., *g*_*M*-1_(*n*)}, which are described by well-defined parameters from a very large and redundant dictionary **D**:

(1) $$x(n)=\sum_{m=0}^{M-1}a_{m}g_{m}(n)+e(n),$$

where *M *is the number of decomposed t-f components, *a*_*m *_is the coefficient, *a*_*m*_*g*_*m*_(*n*) is the *m*-th t-f component and *e*(*n*) is the decomposition residue. Note that, in the MP algorithm, a signal's t-f components are in the form of the basis functions defined by the dictionary.

Although *x*(*n*) can be perfectly approximated using orthonormal functions such as the delta functions, we are interested in sparse approximation. That is, we aim to adaptively choose a finite number (*M*) of basic waveform functions, which are capable of explaining the signal's representative time-frequency features, from a redundant dictionary **D**. An optimal sparse approximation of the signal *x*(*n*) is obtained when the norm of the residue vector *e*(*n*) in (1) is minimized. It is generally achieved by an iterative algorithm as follows:

(2) $${\tilde{g}}^{\left(k\right)}(n)=\underset{g_{m}(n)\in D}{\arg\max}\left\|\langle e_{x}^{\left(k\right)}(n),g_{m}(n)\rangle \right\|,$$

(3) $$e_{x}^{\left(k+1\right)}(n)=e_{x}^{\left(k\right)}(n)-\langle e_{x}^{\left(k\right)}(n),{\tilde{g}}^{\left(k\right)}(n)\rangle {\tilde{g}}^{\left(k\right)}(n).$$

In the initial step, the waveform ${\tilde{g}}^{\left(0\right)}(n)$ with the highest inner product with the signal *x*(*n*) [the initial residue $e_{x}^{\left(0\right)}(n)=x(n)$], which means that it accounts for the largest part of the signal energy, is found from the dictionary **D** to generate the main t-f component $a^{\left(0\right)}{\tilde{g}}^{\left(0\right)}(n)=\langle {\tilde{g}}^{\left(0\right)}(n),e_{x}^{0}(n)\rangle {\tilde{g}}^{\left(0\right)}(n)$. Then, the residual $e_{x}^{\left(1\right)}(n)$ is computed as (3) and ${\tilde{g}}^{\left(1\right)}(n)$ is obtained by matching it to $e_{x}^{\left(1\right)}(n)$ as (2). The two steps are executed iteratively until some stopping criterion is reached. For instance, in this study the iteration will stop when decomposed components account for 99.5% of the signal energy.

The Gabor dictionary was employed in this study because Gabor functions exhibit good time-frequency localizations [14,15]. A Gabor function is expressed as:

(4) $$g(n)=K\cdot e^{-\pi {\left[(n-t)/s\right]}^{\text{2}}}\cos(2\pi f(n-t)+\phi ),$$

where *K *is the normalization parameter to make ||*g*(*n*)|| = 1. In a Gabor function, $e^{-\pi {\left[(n-t)/s\right]}^{\text{2}}}$ is the waveform envelope with center at time *t *and span described by *s*. The parameter *t *is the waveform's latency, which is defined as the time duration from the stimulus onset to the maximum of the waveform envelope. It can be seen that the basic functions used in MP algorithm are generated by dilating (with *s*), translating (with *t*), modulating (with *f*) and phase-shifting (with *φ*) an envelope function (a Gauss function). Therefore, the parameters to determine a Gabor function of (4) include *t *(latency), *f *(frequency), *s *(time span) and *φ *(phase). These parameters, together with *a *(amplitude) of (1), will constitute a parameter vector ***u ***= [*t*, *f*, *s*, *φ*, *a*]^*T *^and will be used to characterize a decomposed t-f component *a*_*m*_*g*_*m*_(*n*).

Because the parameters are chosen from the Gabor dictionary **D**, the ideal size of the dictionary **D **should be infinite in theory to achieve perfect matching results. In practice, the dictionary **D **only includes limited candidate values to avoid heavy computational load, so the precision of the parameters is not high enough. This paper proposes to further refine the parameters by nonlinear least-squares (NLS) algorithm. We notice that, in fact, Eq. (2) is a NLS problem, and it can be solved by the Gauss-Newton method or the Levenberg-Marquardt method. The optimal parameters found by the greedy research can be used as the starting value for the NLS algorithms. By the NLS-based refining, the optimal parameters beyond the scope of **D **can be obtained and they have higher precision than the parameters obtained by the greedy search. The Gaussian-Newton NLS algorithm was adopted in this study and more details regarding the NLS algorithms were referred to [24].

### Identification and classification of t-f components

To further classify the t-f components and identify the stable SEP t-f components, a clustering method based on joint probability density function (PDF) estimation was used. The PDF-based clustering was employed in this study because it can handle the noise (i.e., the spurious and unstable t-f components) well [22]. However, because it is difficult to compute and illustrate PDF in a five-dimensional space defined by the Gabor parameter vector ***u ***= [*t*, *f*, *s*, *φ*, *a*]^*T*^, a dimensionality reduction is first required. In this study, the five features are reduced to three: latency *t*, frequency *f*, and relative energy *ε*. These three features are employed because time-frequency analysis leads to a representation for the signal in the time-frequency-energy space. The energy of a t-f component is calculated as the sum of the squared magnitudes of the t-f component $E_{m}=\sum_{n}{\left|a_{m}g_{m}(n)\right|}^{2}=a_{m}^{2}$. That is, the energy of a t-f component is just the squared amplitude parameter, and the information on span and phase parameters is not included in the energy. Furthermore, the feature "relative energy" is introduced to make t-f components from different SEP signals comparable. For a t-f component decomposed from a SEP signal *x*(*n*), its relative energy was calculated as the ratio between the energy of the t-f component, *E*_*m*_, to the total energy of signal *x*(*n*), *E*_*total*_, i.e., *ε*_*m *_= *E*_*m*_/*E*_*total*_, where $E_{total}=\sum_{n}{\left|x(n)\right|}^{2}$. Accordingly, the joint PDF is calculated in the three-dimensional (3D) space described by the simplified parameter vector ***v ***= [*t*, *f*, *ε*]^*T*^.

Suppose *N *t-f components in total are extracted from 28 rat SEP signals, and each component is described by a parameter vector ***v*_*i *_**= [*t*_*i*_, *f*_*i*_, *ε*_*i*_]^*T*^, *i *= 1, 2, ..., *N*. The joint PDF in the 3D time-frequency-energy space was estimated by the conventional kernel density estimation algorithm using Gaussian kernel as:

(5) $$P(v)=\sum_{i=1}^{N}\frac{\text{1}}{{\text{(2}\pi \text{)}}^{N\text{/2}}\det(\Delta )}e^{\text{-[}{\left(v-v_{i}\right)}^{T}\Delta^{-1}(v-v_{i})]/2},$$

where Δ is the bandwidth matrix to adjust the smoothness of the PDF and det(Δ) is the determinant of Δ. The bandwidth matrix should be carefully selected to avoid an under-smoothed or over-smoothed PDF. In our study, the bandwidth matrix was chosen as a diagonal matrix Δ = diag([*δt*, *δf*, *δε*]), where *δt*, *δf *and *δε *were optimally selected to minimize the asymptotic mean integrated squared error (AMISE) in respective dimension. More details of the kernel density estimation and optimal bandwidth selection can be found in [25].

All the peaks (local maxima with higher density than neighbouring points) in the 3D PDF were detected as potential locations of a cluster (a class of stable t-f components), and the region of a potential cluster is the peak region around one peak and with PDF values greater than *ξ *‧ *P*_*LocalMax*_, where *P*_*LocalMax *_is the local peak value and *ξ *is a threshold parameter between 0 and 1. If the number of t-f components inside one peak region is larger than *η *‧ *N*, where *η *is another threshold between 0 and 1 to determine the minimum occurrence of t-f components to confirm a cluster, these components were clustered into a class of stable SEP t-f component. Otherwise, the t-f components were recognized as unstable or spurious.

Two thresholds *η *and *ξ *work jointly to influence the clustering results. A smaller *η *means that the condition to be a cluster is becoming loose and more clusters can be discovered. A larger *η *tightens the condition so that fewer clusters will be identified. On the other hand, if the threshold *ξ *is larger, the region of a potential cluster will be smaller and fewer t-f components will be contained in the peak region. As a result, fewer clusters will be recognized when *ξ *become larger. On the contrary, a smaller *ξ *yields a broader region covering more t-f components and thus more clusters can be confirmed. However, if *ξ *is too small, these peak regions may overlap each other. In this situation, the peak region with a smaller peak value will be merged into the peak region with a larger peak value, which reduces the number of clusters. In addition, a too wide region implies unwanted large variances for parameters of t-f components in the peak region.

In our study, we first determined the threshold *η*, and *ξ *was chosen based on the given *η*. We believe a stable t-f component of SEP originates from an inherent response to the stimulus and it should occur in each single subject with similar properties. Even if the noise is very large, the stable t-f component should be detected in at least half of subjects. Thus, the threshold *η *was set as *η *= 0.5. As to the threshold *ξ*, its optimal value is difficult to be obtained in an analytical form. We tested and compared a series of values and set *ξ *as *ξ *= 0.5 because this value was capable of identifying a set of stable t-f components having concentrated regions in the feature space. Other threshold values far from *ξ *= 0.5 could not identify any cluster (*ξ *is too large) or could not identify clusters with concentrated regions (*ξ *is too small).

It should be also noted that some t-f components may be wrongly classified because of the dimension reduction. After detailed investigation on the experimental results, we found that a few anomaly values (outliers), which were quite different from data majority, often existed in parameters of clustered t-f components. The components with outlier parameters should be removed from the cluster because they had different nature from most other components in the cluster and they contributed considerably to the large SD values. In this paper, an easily-implemented outlier detection technique, the Grubbs' test (with significance level 0.05), was employed to identify the outliers [26]. If one parameter (or more) of a t-f component is far away from the rest in the cluster, the t-f component was labeled as an "outlier" and was removed from the cluster.

## Results

**Figure 1:**
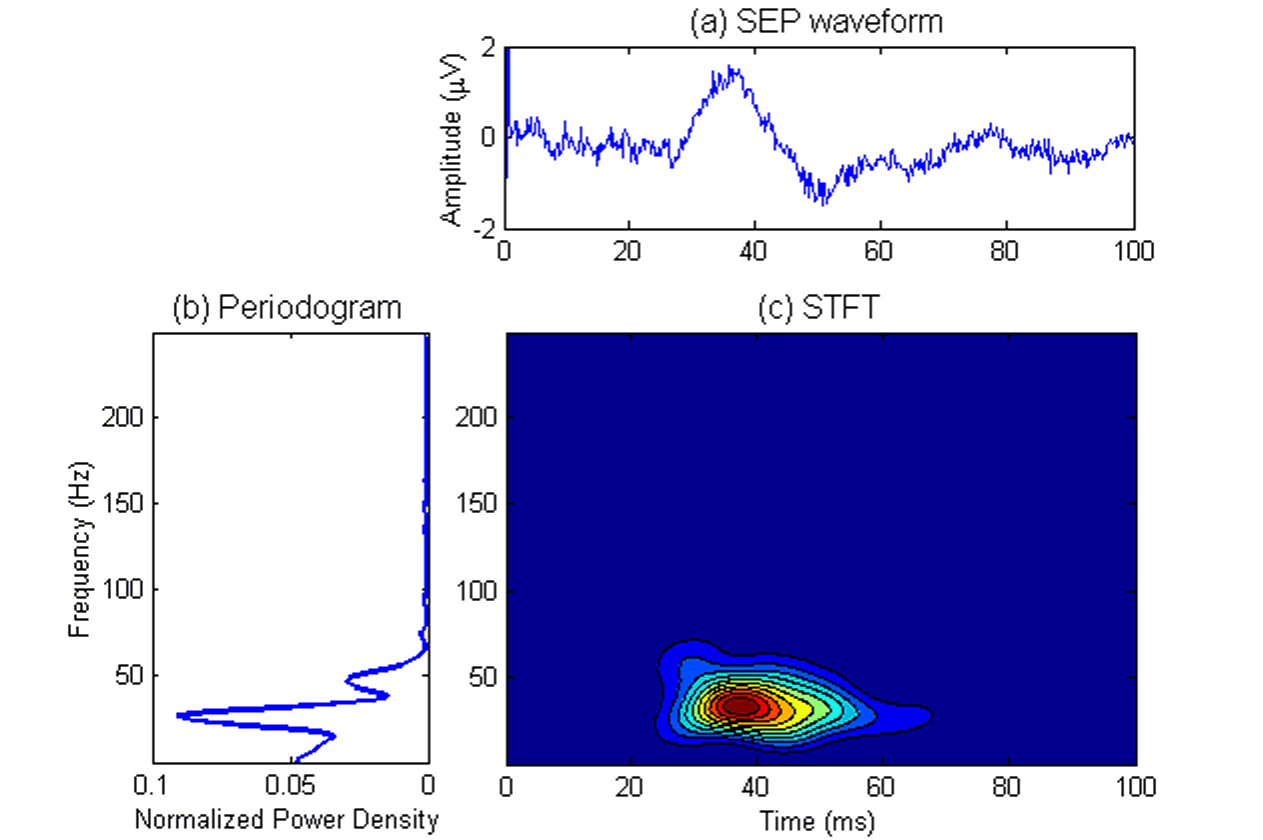
**An example of SEP signal, its periodogram, and its STFT-based TFD**. (a) A typical SEP waveform. (b) Periodogram with 1024-point fast Fourier transform (FFT). (c) STFT with a 40 ms Hanning window.

**Figure 2:**
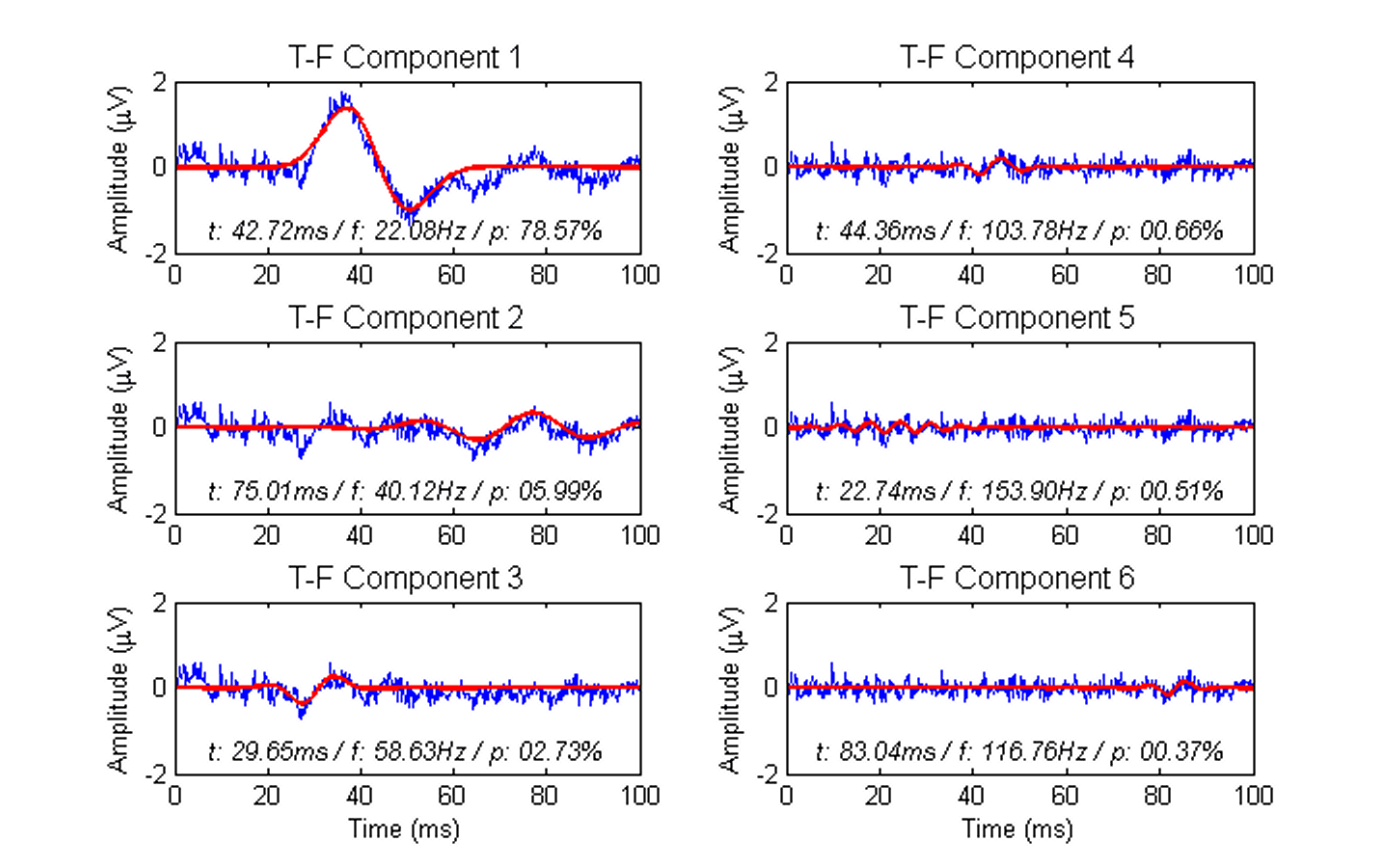
**An illustration of MP decomposition**. Six highest energy t-f components were decomposed from the SEP signal shown in Figure 1 and their parameters are shown as well. Red bold lines indicate the Gabor components and blue thin lines indicate the decomposition residues in previous iteration.

**Figure 3:**
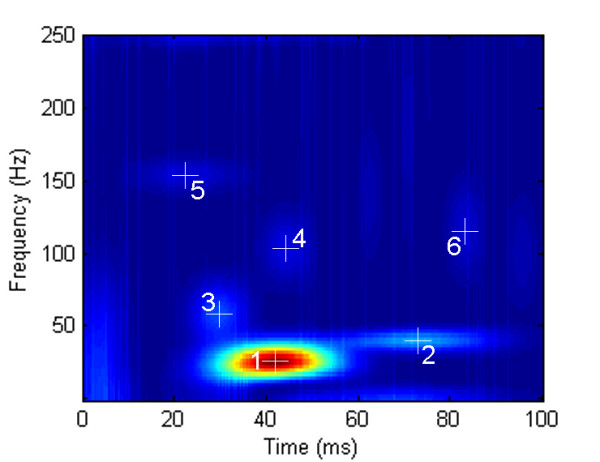
**Time-frequency energy distribution of all t-f components decomposed by MP algorithm**. The Wigner-Ville distributions were calculated from all the t-f components decomposed from the SEP signal in Figure 1. The serial numbers of the t-f components shown in Figure 2 are labeled and the cross signs indicate the latency-frequency positions of these t-f components in the t-f domain.

A typical SEP signal in rat and its TFD based on a 40 ms Hanning-windowed STFT are demonstrated in Figure 1. There is only one dominant peak illustrated in the TFD and other minute t-f subcomponents can hardly be identified. Unlike the STFT method, the MP algorithm does not directly present an energy distribution in t-f domain but decomposes the signal into a series of components described by t-f parameters. Figure 2 shows a set of t-f components decomposed from the SEP signal in Figure 1. We also calculated the Wigner-Ville distributions (WVD) of all the t-f components decomposed from the signal in Figure 1 and gave an energy distribution of all these t-f components in the time-frequency space [15,16], as shown in Figure 3.

**Figure 4:**
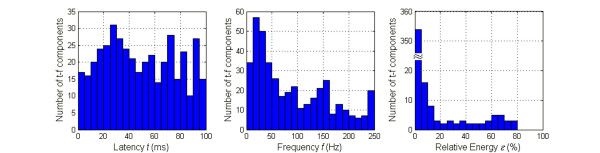
**Histograms of MP parameters from 28 SEP signals**. The total number of t-f components is 395. The width of latency bin is 5 ms; the width of frequency bin is 12.5 Hz; the width of relative energy bin is 5%.

In this study, MP decomposition was performed until decomposed components explained 99.5% of the energy of each signal. Those t-f components with too low frequency (*f *< 1 Hz) or too short time span (*s *< 2 ms) were discarded as spurious components. As a result, a total of 395 t-f components were decomposed from 28 SEP signals and were used for further statistical analysis. Figure 4 represents the histograms of MP parameters (*t*, *f*, *ℇ*) for all 395 decomposed components. Figure 4(a) indicates a main peak located around time instant 30 ms and several small peaks after 50 ms. Histogram of Figure 4(b) shows that the majority of components had a frequency ranging from 10 to 40 Hz. It can also be seen from Figure 4(c) that the energy of most SEP t-f components was very small. In fact, around 80% of components (312/395 components) had relative energy values less than 2%. Although Figure 4 indicates some useful information on the distribution of SEP t-f components, this information is obscure and may be inaccurate because there is a great deal of unstable and spurious t-f components included.

**Figure 5:**
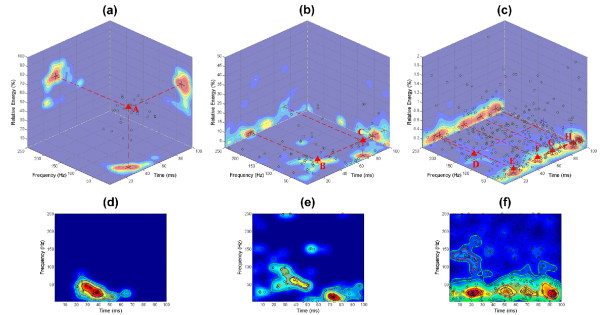
**Distributions of SEP t-f components**. (a)-(c) Distributions of high-energy, middle-energy, and low-energy t-f components in time-frequency-energy space. (d)-(f) Distributions of high-energy, middle-energy, and low-energy t-f components in time-frequency space and peak regions under different values of region threshold. The projections of 3D distributions on time-frequency/time-energy/frequency-energy domains are also illustrated in (a)-(c). The black circles denote the t-f components, and the red triangles indicate the clusters. The blue, red, green lines in (d)-(f) indicate the peak regions when *ξ *= 0.75, 0.5, and 0.25, respectively.

**Figure 6:**
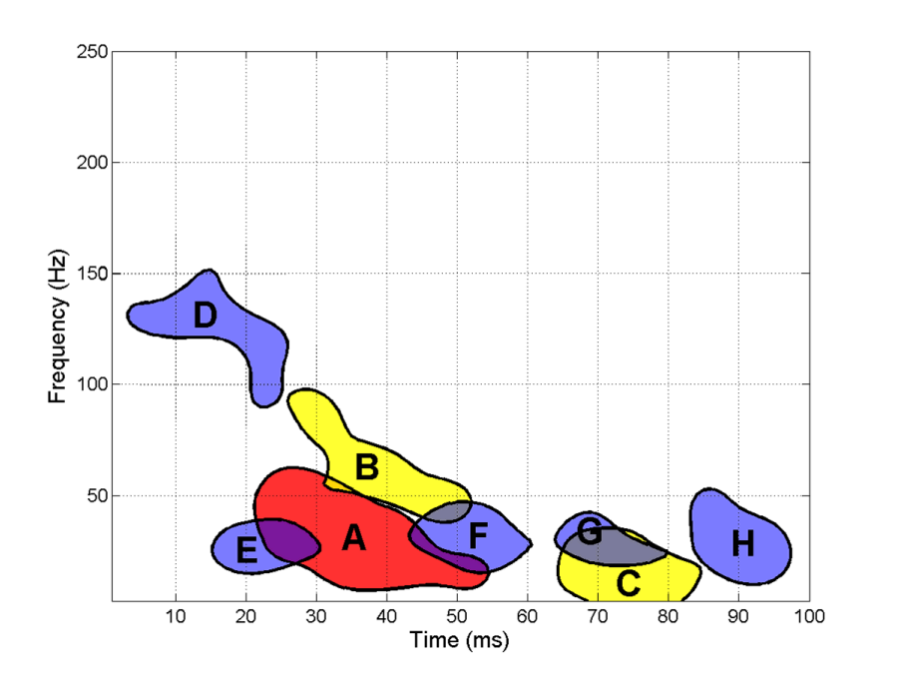
**Distribution of latency and frequency of stable SEP t-f components**. Based on their relative energy, the seven stable t-f components are distinguished by color. Red color denotes the high-energy t-f components, yellow denotes the middle-energy t-f components, and blue denotes the low-energy t-f components.

To obtain easily-identified distribution patterns of stable t-f components, the joint PDF in the 3D time-frequency-energy space is required to be calculated. First, these decomposed components were classified into three categories regarding the relative energy: 1) the component with the highest energy in each signal was defined as the high-energy component, i.e., only one high energy component per each subject 2) other components, except the high-energy component, with relative energy greater than 2% were defined as middle-energy components; 3) the remain components were defined as low-energy components. The joint PDFs in time-frequency-energy space for the three types of t-f components were calculated using Gaussian kernel-based density estimation and are illustrated in Figure 5. It can be seen that the distribution patterns of the components scattered in the 3D space are difficult to reveal without the help of PDF estimates. Based on the joint PDF estimates and clustering, eight classes of stable t-f components were identified and they were: high-energy component A, middle-energy components B and C, and low-energy components D, E, F, G and H. Class A is the main SEP t-f component, while the middle-energy and low-energy components (Classes B – H) are regarded as t-f subcomponents. The locations of these identifiable stable t-f components are marked in the 3D space and the two-dimensional projections, as shown in Figure 5. By projecting the 3D peak areas of stable t-f components onto the t-f domain, an approximate distribution map of the latencies and frequencies of the eight classes of stable t-f components was obtained, as shown in Figure 6.

The mean, standard deviation (SD), coefficient of variation (CV), and appearances (in how many rat subjects this class of stable t-f component could be identified) of the eight classes of stable components are exhibited in Table 1. The CV values were calculated as CV = 100‧SD/Mean and they were used here to measure and compare the variability between different sets of parameters. Because CV is not suitable for parameters having negative values and with mean values close to zero, the CV values for phase were not listed in Table 1. For each parameter, the average CVs of all eight classes of t-f components were 23 (latency), 39 (frequency), 62 (span), and 54 (amplitude). We can see that the span parameter has the largest variability because it was not directly used for clustering. On the other hand, for each class of stable t-f components, the average CVs of all parameters were 41 (A), 41 (B), 39 (C), 52 (D), 44 (E), 45 (F), 45 (G), and 50 (H). That is, the CVs of Classes D-H are relatively greater than those of A-C.

**Table 1:** Parameters and appearances of eight classes of stable SEP t-f components. The values of parameters are given as mean ± SD (CV).

**Class**	**A**	**B**	**C**	**D**
Latency *t *(ms)	37.35 ± 10.60 (28)	35.34 ± 9.33 (26)	75.83 ± 7.03 (9)	15.37 ± 10.61 (69)

Frequency *f *(Hz)	30.04 ± 12.54 (42)	64.63 ± 21.27 (33)	20.75 ± 9.17 (44)	126.31 ± 25.04 (20)

Time span *s *(ms)	41.03 ± 17.69 (43)	29.72 ± 14.65 (49)	52.46 ± 32.47 (62)	25.93 ± 15.77 (61)

Phase *φ *(rad)	0.07 ± 0.02 (--)	-0.05 ± 0.02 (--)	-0.01 ± 0.03 (--)	0.01 ± 0.02 (--)

Amplitude *a *(*μ*V)	2.18 ± 1.14 (52)	0.50 ± 0.28 (56)	0.75 ± 0.30 (40)	0.10 ± 0.06 (60)

Relative Energy (%)	60.67 ± 12.72 (21)	7.50 ± 5.03 (67)	10.03 ± 4.80 (48)	0.43 ± 0.20 (47)

Appearances	25	18	14	15

Appearance Rate (%)	89.29	64.29	50.00	53.57

**Class**	**E**	**F**	**G**	**H**

Latency *t *(ms)	24.38 ± 6.75 (28)	48.12 ± 5.22 (11)	71.46 ± 6.05 (8)	89.63 ± 5.36 (6)

Frequency *f *(Hz)	30.60 ± 11.87 (39)	35.04 ± 12.28 (35)	28.77 ± 14.10 (49)	28.29 ± 13.87 (49)

Time span *s *(ms)	21.96 ± 14.67 (67)	11.90 ± 8.78 (74)	18.15 ± 12.81 (71)	13.21 ± 9.66 (73)

Phase *φ *(rad)	-0.02 ± 0.04 (--)	-0.08 ± 0.02 (--)	-0.03 ± 0.06 (--)	-0.04 ± 0.03 (--)

Amplitude *a *(*μ*V)	0.07 ± 0.03 (43)	0.13 ± 0.08 (62)	0.06 ± 0.03 (50)	0.11 ± 0.08 (73)

Relative Energy (%)	0.12 ± 0.10 (83)	0.20 ± 0.13 (65)	0.09 ± 0.08 (89)	0.25 ± 0.15 (60)

Appearances	16	14	17	15

Appearance Rate (%)	57.14	50.00	60.71	53.57

Furthermore, we employed the Kolmogorov-Smirnov test [27], which is a popular normality test, to see whether these parameters are Gaussian distributed. The results show that only the phase parameters of Classes E-H cannot pass the normality test (rejection level 0.05). As a result, except the phase parameters for E-H, other parameters for any class of t-f components can be considered as Gaussian distributed.

**Figure 7:**
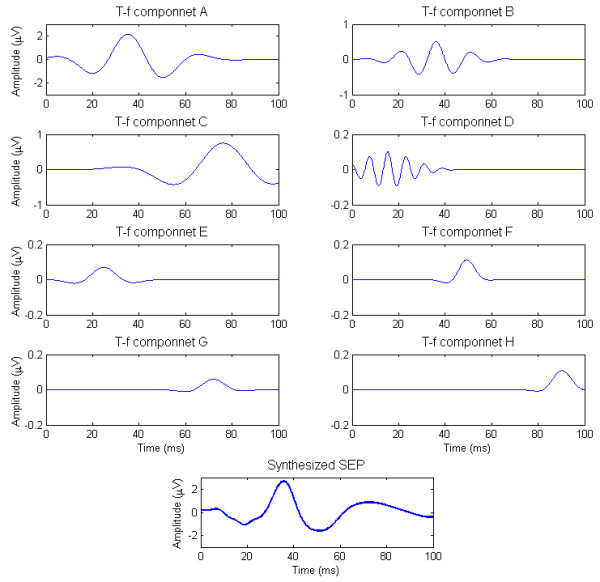
**A synthesized rat SEP signal and its t-f components**. Waveforms of eight classes of stable t-f components were synthesized using the mean values of parameters for t-f components in Table R1. The typical SEP waveform was obtained as the sum of eight synthesized t-f components. These waveforms are plotted in different scales.

Based on the mean values of parameters of each class in Table 1, a typical rat SEP signal and its t-f components were synthesized and they were illustrated in Figure 7. It can be seen from Figure 7 that, 1) the main t-f component A is approximately a W-shaped waveform comprising two troughs separated by a main ridge; 2) Class B has a similar latency with A but its frequency is higher; 3) Class C is a low-frequency long-latency component; 4) Class D is a high-frequency component spreading over the whole time range with diminishing magnitudes; 5) the low-energy t-f components D-H are a string of low-frequency long-duration components.

**Figure 8:**
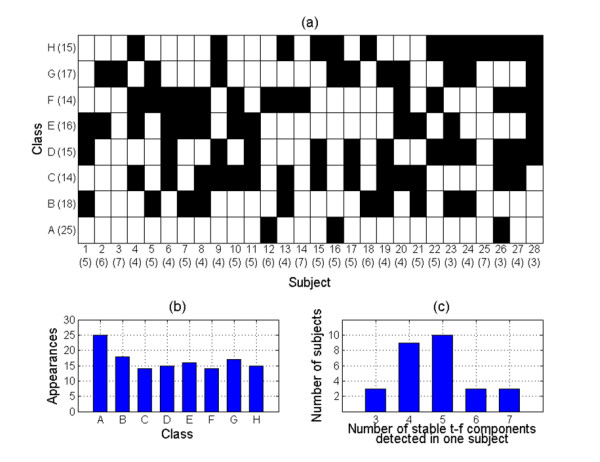
**Appearances of stable t-f components in each individual rat subject**. (a) Stable t-f components detected in each individual subject: a white block means the corresponding t-f component can be identified in this subject, while a black block means this component cannot be found in this subject. The numbers in brackets after the component names are the appearances of components, while the numbers in brackets below the subject number are the number of components detected in this subject. (b) Histogram of appearances of stable t-f components. (c) Histogram of number of stable t-f components detected in one subject.

Furthermore, Figure 8(a) and 8(b) illustrate the appearances of the eight classes of stable t-f components in each individual rat subject and the histogram of the appearances of stable components, respectively. It can be seen from Figure 8(a) that not all stable t-f components could be found in one individual rat subject. At most seven classes of stable t-f components (one main component and six subcomponents) could be identified from one SEP signal, which happened in 3/28 rat subjects. The worst case is that there were 3/28 subjects in which only three classes of stable components were identified. Figure 8(c) represents a histogram of the number of stable t-f components identified in one individual subject.

## Discussion

In this paper, we employed the MP algorithm to decompose a SEP signal into a series of t-f components, which were in the form of Gabor functions and defined by 5 parameters. Dimension reduction is a must because it is difficult to conduct a clustering in a five-dimensional space, which is known as the "curse of dimensionality" [22]. Because the TFA yields a time-frequency-energy representation for the signal, we reduced the parameters to latency, frequency, and relative energy. Although a clustering in two-dimensional time-frequency space is easier, it may underestimate the number of clusters. Because dimension reduction may cause wrong clustering results, outlier detection was employed to refine the results.

Eight classes of stable SEP t-f components were identified in our study, and the complex response patterns of a "perfect" SEP recording with all identifiable stable t-f components were illustrated in Figure 6 and Figure 7. After a stimulus, a high-frequency (*f *> 100 Hz) low-energy (*ℇ *< 0.5%) subcomponent D will occur first (*t *< 25 ms). Then, the main t-f component A and a middle-frequency (50 <*f *< 100 Hz) middle-energy (2% <*ℇ *< 10%) subcomponent B will appear simultaneously (25 <*t *< 50 ms), which suggests that B is an affiliated or associated t-f component of the main component. Another middle-energy (5% <*ℇ *< 20%) subcomponent C with a low frequency (*f *< 50 Hz) responds much later (65 <*t *< 85 ms), and it may be a subsequent response evoked by preceding strong nerve response. In addition, a series of low-frequency (*f *< 50 Hz) low-energy (*ℇ *< 0.5%) subcomponents (E, F, G and H) can be found in the whole response.

The existence of the main t-f component (Class A) in SEP of a normal subject is clear and certain, and it has also been validated by other TFA methods such as STFT [9-12]. As to other classes of components, their existences have not been reported before but are successfully identified by the PDF-based clustering. However, the thresholds used in the PDF-based clustering were determined in a trial and error fashion, and there is no gold standard to validate the clustering results. Therefore, we need further check the statistical properties of these components and give reasonable explanations or assumptions for the origins of these t-f components. As seen from the results in previous section, Class B and Class C have clear distribution patterns in the feature space, specific waveforms, and relatively small SD and CV values. Therefore, t-f components B and C should be inherent responses in rats' SEP.

The possibility that five classes of low-energy components D-H are noise cannot be ruled out, and the judgment was supported by the following facts: 1) these t-f components have relatively large SD and CV values; 2) for classes E-H, almost all parameters, with the exception of latency, exhibit similar statistical characteristics; 3) if a lower threshold *ξ *was set in the PDF-based clustering, D-H may be merged into a large region. In fact, it can be seen from Figure 5(c) that almost the whole PDF region with frequency below 50 Hz had large PDF values. If the t-f components D-H are really originated from noise, class D may be caused by stimulus artifacts, while the long-duration and consistent electrophysiological interference, such as the alpha (8–12 Hz) and beta (12–30 Hz) waves of EEG, may be the sources of classes E-H.

Overall, the existences of some identifiable t-f subcomponents are still uncertain and their physiological basis is also vague, although some valuable studies were carried out [3,4,28-30]. Therefore, in future work we need use new animal model and experimental protocol to disclose the origins of these subcomponents.

Except for the identifiable eight classes of stable t-f components, other spurious and unstable components decomposed by the MP algorithm were considered to be caused by the electrophysiological or other noise and have little significance. Especially, the components at harmonics of 50 Hz and spanning almost the whole signal should be due to power-line interference. Possibly, some unstable t-f components were true physiological phenomena, but their appearances were too low to be recognized as an inherent and common component of SEP. These unstable t-f components can be used in future to study the inter-subjective variability of SEP.

From Table 1 and Figure 8, we can see that: 1) no t-f subcomponent could be successfully identified from every SEP signal under study, 2) no SEP signal contained all seven stable t-f subcomponents and 3) some parameters, especially the time span, phase, and amplitude, exhibited very large variabilities (the ratio of SD and mean). These problems may be due to information loss during data acquisition and the limitations of the current MP algorithm and clustering technique.

SEP signals used in our experiments were collected using a 12-bit data acquisition card so that the data precision is low. High-precision data acquisition cards should be adopted in future to collect higher bit-depth SEP data. Moreover, the SEP signals were obtained by ensemble average of 100 SEP recordings and some minute subcomponents may be smoothed out during the average operation, because latency shifts are very common for conducting potentials [4]. On the other hand, if the experiments are conducted using single trial SEP, the large amount of noise in single-trial SEP may produce many more spurious components. With the development of fast and accurate SEP extraction techniques, it is expected that more subcomponents can be extracted and identified in high-SNR single trial SEP recording.

Regarding the MP algorithm, Gabor functions are adopted in this paper as the basic components. However, Gabor functions cannot satisfactorily describe a component with time-varying frequency or asymmetric envelope. Other complex dictionaries, such as the chirplet dictionary [19,20], can also be used in the MP decomposition at the expense of greatly increased computational load. As to the clustering, the PDF estimation and feature extraction in a higher-dimensional space can be adopted in future. Other clustering techniques can also be under consideration.

Unfortunately, SEP signals recorded in operating theaters are always contaminated with heavy electrophysiological activity and other noise, which increases the signal variability and makes latency and amplitude measurement difficult and inaccurate, especially for the detailed components [2]. This study used SEP data from rats for the TFA components study because the SEP signals from rats provide higher intensity and are easier to extract t-f components than SEP from humans. The results of this study can be easily carried on further with localized injury to the spinal cord. The eight stable SEP t-f components identified in the current study and the associated parameters are based on normal rat subjects without any spinal cord injury. When injury occurs, these t-f components and their parameters may indicate certain change patterns. Investigation on the change of SEP t-f components during different surgical stages may be of clinical value in intraoperative spinal cord monitoring.

## Conclusion

Overall, this study identified a series of stable SEP t-f components conveying important temporal and spectral information on SEP in rats. First, the high-resolution MP method decomposes the SEP signal into a number of t-f components. A joint PDF estimate is followed to obtain the distribution of these t-f components in the high-dimensional space defined by component parameters. Finally, the high-density areas in the high-dimensional space are detected as the locations of stable SEP t-f components. The identified SEP t-f components may contribute to understanding complex response patterns and physiological origins of SEP.

## Competing interests

The authors declare that they have no competing interests.

## Authors' contributions

ZGZ carried out the signal processing work, performed the statistical analysis, and drafted the manuscript. JLY conducted animal experiments and interpretation. DKL conceived the overall research direction and designed the experiments. SCC participated in the signal analysis aspects of the project. YH conceived and outlined the overall research direction, contributed to design and project coordination, and helped to draft the manuscript. All authors read and approved the final manuscript.
